# Building up Graphene-Based Conductive Polymer Composite Thin Films Using Reduced Graphene Oxide Prepared by ****γ****-Ray Irradiation

**DOI:** 10.1155/2013/954324

**Published:** 2013-09-19

**Authors:** Siyuan Xie, Bowu Zhang, Chunlei Wang, Ziqiang Wang, Linfan Li, Jingye Li

**Affiliations:** ^1^TMSR Research Center and CAS Key Lab of Nuclear Radiation and Nuclear Energy Technology, Shanghai Institute of Applied Physics, Chinese Academy of Sciences, Shanghai 201800, China; ^2^University of the Chinese Academy of Sciences, Beijing 100049, China

## Abstract

In this paper, reduced graphene oxide (RGO) was prepared by means of **γ**-ray irradiation of graphene oxide (GO) in a water/ethanol mix solution, and we investigated the influence of reaction parameters, including ethanol concentration, absorbed dose, and dose rate during the irradiation. Due to the good dispersibility of the RGO in the mix solution, we built up flexible and conductive composite films based on the RGO and polymeric matrix through facile vacuum filtration and polymer coating. The electrical and optical properties of the obtained composite films were tested, showing good electrical conductivity with visible transmittance but strong ultraviolet absorbance.

## 1. Introduction

Graphene has recently attracted much interest in the scientific community because of its distinctive two-dimensional structure and physicchemical properties [1–4]. Different from other 3-dimension substances, graphene has a zero bandgap structure, where electrons and holes are closely connected through a relativistic Dirac equation [5]. The energy bands are cones *ε*1; 2(*p*) = ±*νp* at the *K* points in the 2D Brillouin zone with the constant velocity parameter *ν* = 10^8^ cm/s [6]. As reported previously, the sheet resistance of a single-layer undoped graphene is around 6000 Ω/sq [7] coupled with about 97.7% of white light transmittance [8]. Moreover, the sheet resistance of graphene can be reduced to even 50 Ω/sq by chemical doping [9]. Therefore, graphene is increasingly seen as a promising alternative to replace traditional transparent conductive materials such as indium tin oxide (ITO) [10], especially when considering its other advantages, such as mechanical strength, flexibility, chemical stability, and low cost.

 Until now, in cases of high-quality transparent conductive film for displays, graphene is almost always prepared by a chemical vapor deposition (CVD) method [11–13] because the properties of products are close to pristine grapheme, and the stack of sheets is relatively controllable. This approach always requires high-quality and specially treated metal or SiC/Si wafer as the graphene growth substrate. After deposition, the substrate should be removed, and the sheets were transferred onto other arbitrary substrates. Therefore, cost and technique thresholds of this approach are limitations to industrial application. Li et al. [12] demonstrated that graphene dispersions with concentrations up to ~0.01 mg/mL can be produced by dispersion and exfoliation of graphite in organic solvents such as N-methyl-pyrrolidone with the assistant of ultrasonication. Therefore, a graphene-based liquid crystal device has been prepared through spray coating of the graphene dispersion mentioned above [7]. This solution processing of graphene opens up a range of potential large-area applications, from device and sensor fabrication to liquid-phase chemistry. However, the yield and dispersion concentration is very low, and the films made from such dispersions are not continuous [12]. 

Although the electrical properties do not rival those of the exfoliated natural graphite, graphene produced from a reduction of graphene oxide (GO) layers by thermal or chemical treatment can produce large quantities of reduced graphene oxide (RGO) platelets that can be formed into thin films, which makes it an option for certain applications, such as antistatic coatings and semitransparent electric circuits. 

There are two strategies to prepare graphene-based conductive film from this approach. The first is by having GO film previously prepared by vacuum filtration and subsequent thermal treatment [14] or chemical reduction coupling with heat annealing [15]. The second is a chemical reduction of GO in solution and then assembly to films by filtration. Generally, the dispersibility of GO after reduction is very poor and prone to aggregation, which is disadvantageous to the building up of continuous graphene films. 

In our previous work, we reported that GO dispersions in ethanol/water irradiated by *γ*-ray under a N_2_ atmosphere are not only highly reduced, but also well dispersed in pure ethanol or ethanol/water mix solutions [16]. Herein, we prepare conductive RGO@polymer films via vacuum filtration and polymer coating (cast or spin coating) using the dispersions of the RGO by radiation-induced reduction.

## 2. Experimental

### 2.1. Chemicals and Materials

 Graphite powder (GP), ethanol, and acetone were purchased from Sinopharm Chemical Reagent Co., Ltd. (Shanghai, China), polyvinyl alcohol (PVA-1788, average Mw *≈* 22000) was purchased from Aladdin Reagent Database Inc. (Shanghai, China), and Polydimethylsiloxane (PDMS) precursor and curing agent were purchased from SYLGARD Dow Corning. All reagents were used directly without further purification. Milli-Q water was used for all experiments. Commercial polyvinylidene difluoride (PVDF) membrane and acetyl cellulose membrane with 0.45 *μ*m pore-sizes were used for filtration.

### 2.2. Radiation Facility

 The *γ*-ray irradiation is performed in a ^60^Co irradiation facility where in the source cell, the 24 rod-like cobalt units are placed in a circle with a total activity of 100,000 Ci. The dose is determined with potassium (silver) dichromate dosimetry system according to JJF1018-90 national standard of China and is measured twice a year. The measured results are compared with the test outcome by the National Institute of Metrology, China, and the deviation is −2.3% (the deviation is allowed in the range of ±5% based on JJF1018-90 national standard). In the experiments, the different dose rates are obtained by putting samples in different radial regions, where the dose rate in different radius is calculated according to the formula given in JJF1018-90 national standard.

### 2.3. Preparation of RGO

 GO was synthesized from the commercial graphite power by a modified Hummer's method as previously reported [17]. First, the GO is dispersed in Milli-Q water by ultrasonication. Then, alcohol is added to the GO dispersion to form GO water/alcohol mixtures with different concentrations of alcohol. The concentration of GO in the resulting mixture is about 0.5 mg/mL. After feeding nitrogen for 15 min, the prepared mixture is sealed and irradiated for a preset absorbed dose and dose rate in the ^60^Co source cell at room temperature. Then, the irradiated mixture is diluted to 0.1 mg/mL by alcohol for further use.

### 2.4. Preparation of RGO@Polymer Films

**Figure 1:**
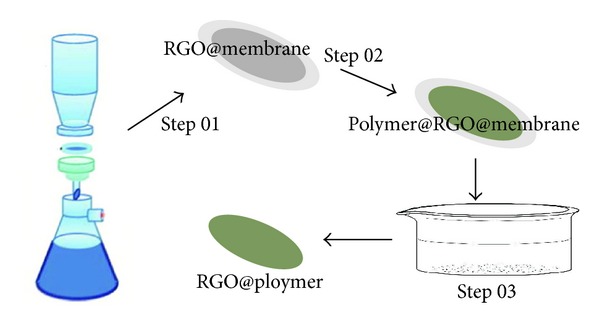
Schematic diagram of the preparation of RGO@polymer composite films.

Figure 1 illustrates the specific procedures for RGO@polymer composite film preparation. Firstly, a RGO film is deposited on a cellulose acetate membrane with 0.45 *μ*m of pore size by vacuum-filtration with the above RGO dispersion (Step 01). The thickness of each RGO film is controlled by adjusting the addition of the RGO dispersion. After drying for one hour in a 60°C oven, the frontal surface of the fabricated film is coated with PVA by spin coating or PDMS by cast coating (Step 02). Note that the PVA solution consists of 15% of mass fraction in Milli-Q water and PDMS precursor mixed with curing agent is prepared at 10 : 1 of mass ratio. The polymer coated film is then dried at 80°C for 30 minutes, and then RGO@polymer films are obtained by removing the cellulose acetate membrane in acetone in the procedure as reported by Wu et al. (Step 03) [18].

### 2.5. Characterizations

 The UV-Vis absorption spectra were obtained on a SCINO PDA S-3100 UV-Visible spectrophotometer using alcohol as solvent. FT-IR spectra were taken by scanning the free-standing GO or RGO paper with the transmission module of a Thermo Nicolet Avatar 370 FTIR spectrometer. The XPS spectra of GO and RGO samples, which had been dried in vacuum oven at 60°C for 24 h, were recorded with a SHIMADZU Kratos AXIS Ultra DLD XPS instrument equipped with a monochromated Al Ka X-ray source. Elemental analysis was carried out with an Elementar Vario EL III elemental analyzer by combustion of RGO and GO power at 950°C. All samples were dried at 80°C in a vacuum oven for 24 h before testing. The optical transmittance was tested on a HITACHI U-3010 spectrophotometer, and the electrical properties of polymer/RGO films were measured using a ST2253 digital four-point probe instrument (Su Zhou Jingge Electronic Co. Ltd., Suzhou, China).

## 3. Results and Discussion

### 3.1. The Radiation Reduction of GO

**Figure 2:**
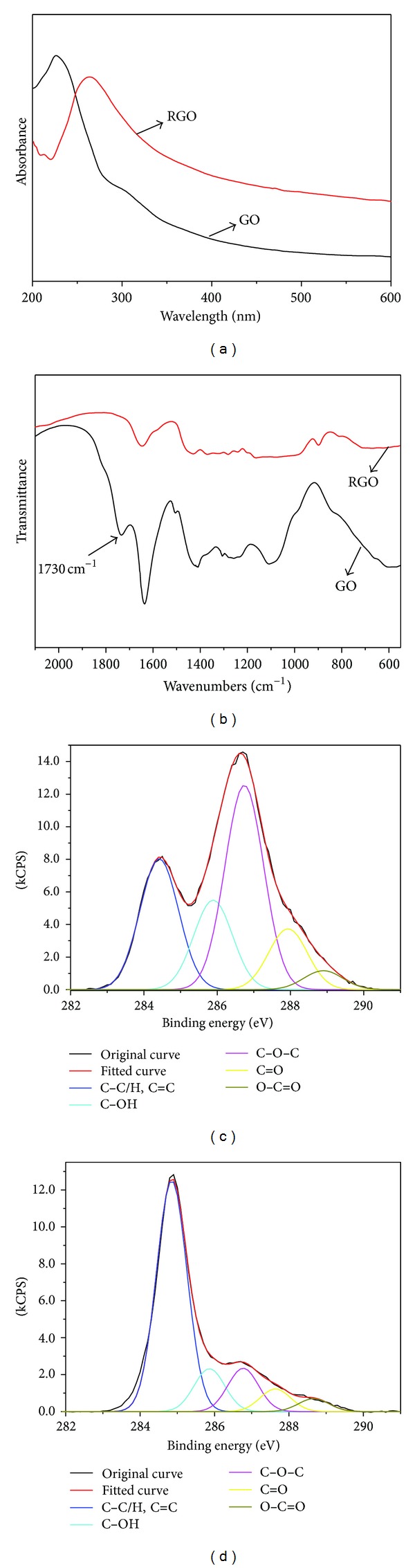
(a) UV-Vis spectra of GO dispersion in ethanol/water before and after *γ*-irradiation; (b) FT-IR spectra of starting GO and RGO measured on transmission mode as thin paper form; XPS C1s spectra of (c) starting GO and (d) RGO obtained by irradiating 34 kGy in 70% ethanol/water at 2 kGy/h under N_2_ atmosphere.

 As reported in our previous work, *γ*-ray induced reduction is a very effective method for the preparation of RGO in solutions [17]. Here, we confirmed the reduction by UV-Vis spectra, FT-IR spectra, and XPS analysis.  Figure 2(a) shows that the maximum absorption peak at 226 nm (*n* → π* transitions of aromatic C=C bonds) of GO is red-shifted to 268 nm after *γ*-ray irradiation in ethanol/water solution under nitrogen atmosphere, which contributes to the restoration of *π*-conjugated network within the graphene sheets [19]. Additionally, the attenuating absorption of shoulder peak around 290 nm (*n* → π* transition of C=O bonds) also indicates the removal of oxygen-contained groups on GO sheets in this process. The disappearance of the characteristic peak of C=O at 1730 cm^−1^ in the FT-IR spectra of RGO (Figure 2(b)) provides another proof for the reduction effect. Furthermore, the appearance of peaks distributed at 1400–1670 cm^−1^ indicates that the aromatic skeletons have been reconstructed in RGO [17], which correspond with the results of the UV-Vis spectra analysis. The reduction effect of GO was additionally investigated on an element level by XPS analysis of carbon. Figures 2(c) and 2(d) show the C1s spectra of starting GO and RGO samples, in which the fraction of carbon containing oxygen is cut down, and it is rational to conclude that GO is reduced. 

### 3.2. The Influence of Reaction Parameters on Reduction

 To evaluate the reduction level of obtained RGO, we determined the C/O ratio of samples through elemental analysis, which is calculated according to the following formula [17]: C/O = 16C%/12O%, while the C% and O% are the mass fractions of carbon and oxygen in the sample.

**Figure 3:**
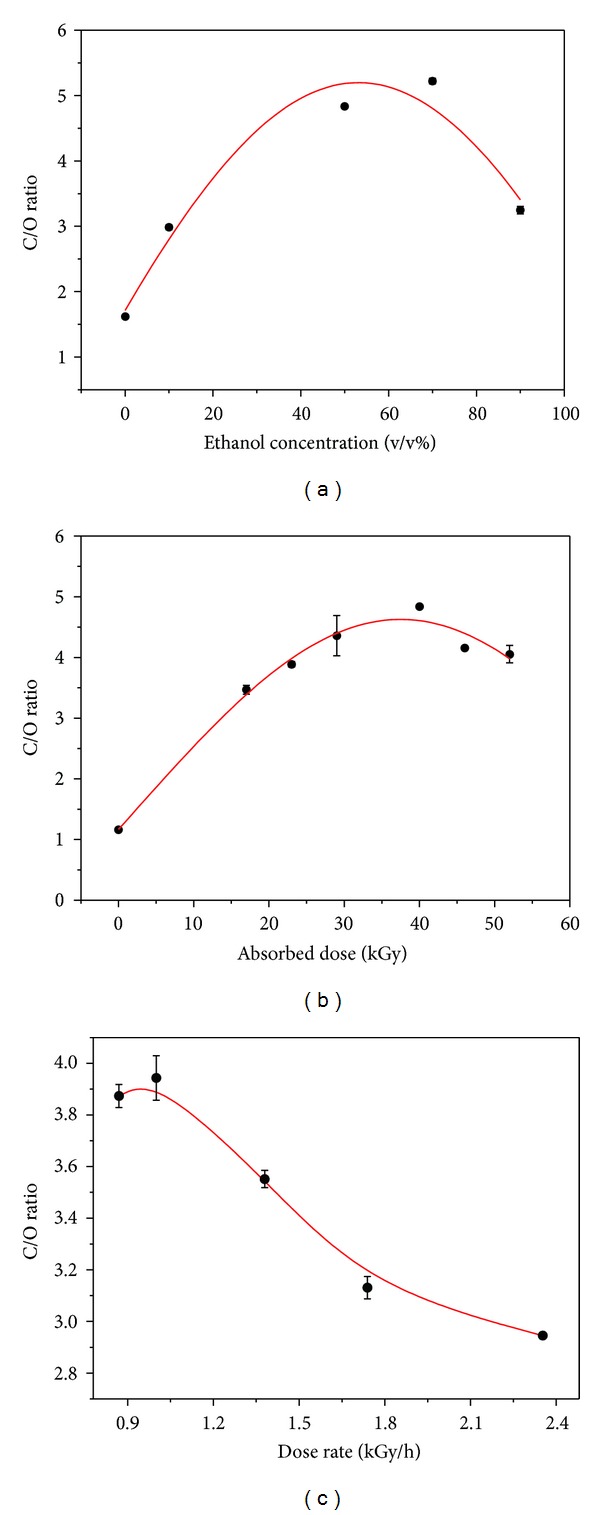
Plots of the C/O ratio of RGO obtained with reaction parameters: (a) ethanol concentration, (b) adsorbed dose, and (c) dose rate.

Figure 3(a) shows that the C/O ratio is a parabolic relationship with the ethanol concentration of the mixture. Increasing ethanol concentration results in the increase of the C/O ratio when it is at low concentration level. This can be attributed to more oxidative radicals eliminated by the adding of ethanol [17, 20]. However, when the concentration of ethanol exceeds 70%, the reduction decreased. As we know, radical reaction is a diffusion-controlled reaction because of radicals' high chemical reactivity [21]. Accordingly, the good dispersed state of reactants in a system is very critical to reaction efficiency. Unfortunately, GO is poorly soluble in ethanol [22] compared with water. Therefore, the excess ethanol depressed the dispersive level of the GO in the reaction system and reduced the chances of reductive radical contact with GO sheets, which knocked down the reduction effect.  Figure 3(b) depicts the influence of absorbed dose on reduction of GO, which shows that C/O ratio rising as absorbed dose increases. It is easy to understand that increasing absorbed dose is helpful to produce more radiolysis species—reductive radicals and hydrated electron—which is the principal reason for GO reduction by irradiation. However, an extremely high absorbed dose could destroy the valence bonds of the graphite layer and cause it to react with other molecules [23], which results in reducing the C/O ratio. The dose rate is also influenced by the reduction effect. As Figure 3(c) shows, a lower dose rate led to a higher reduction, while a higher dose rate obtained a lower reduction level. This would be because the higher dose rate results in an overly fast local reaction with GO, which increases the GO's aggregation and hinders the diffusion of radicals to the sheets. 

### 3.3. Characterization of RGO@Polymer Composite Film

**Figure 4:**
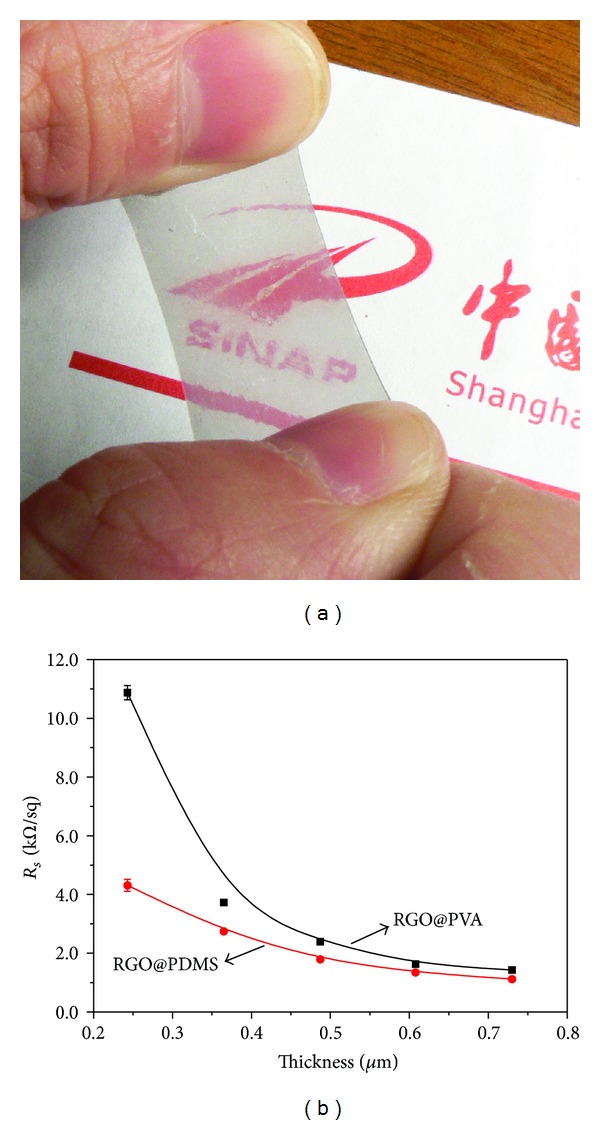
Photograph of (a) RGO@PDMS composite film, (b) the relationship of sheet resistances with the thickness of RGO layer of the composite films.

 As the photographs show in Figure 4(a), RGO@polymer composite film is flexible and optically transparent.  Figure 4(b) shows that the sheet resistance of RGO@polymer films is much higher relative to other graphene-based conductive materials [24]. It is very comparable, even superior, to that of electronic material prepared by depositing GO film on a transparent substrate and subsequent chemical reducing routes which have been reported by Eda et al. [25].

 We further measured the electrical conductivity of films with different thicknesses of RGO on PVA and PDMS substrates, which are shown in Figure 4(b). The thicknesses of these RGO films were calculated by linear relation when we measured the thickness of 30 mL filtration volume with spiral micrometer. As we know, the film of GO or RGO prepared by vacuum filtration is assembled by sheet overlap. When the thickness of GO or RGO is low, there would be some gaps or rifts in the film, which result in its electrical conductivity being lower than the bulk. Here, we also consider the permeation of polymer fluid into the rifts of the RGO film as also a factor that makes the conductivity of RGO@polymer composite films poorer. This conclusion can be rationally demonstrated by comparing the conductivity of RGO@PDMS and RGO@PVA films. PDMS solution is more viscous than PVA solution, and its permeation into RGO film is weaker. Therefore, at the same thickness of RGO, the conductivity of RGO@PDMS is higher than that of RGO@PVA. As the thickness of RGO increases, the gaps and rifts decrease, and the permeation weakens. Therefore, the conductivity of RGO@PDMS and RGO@PVA becomes close.

**Figure 5:**
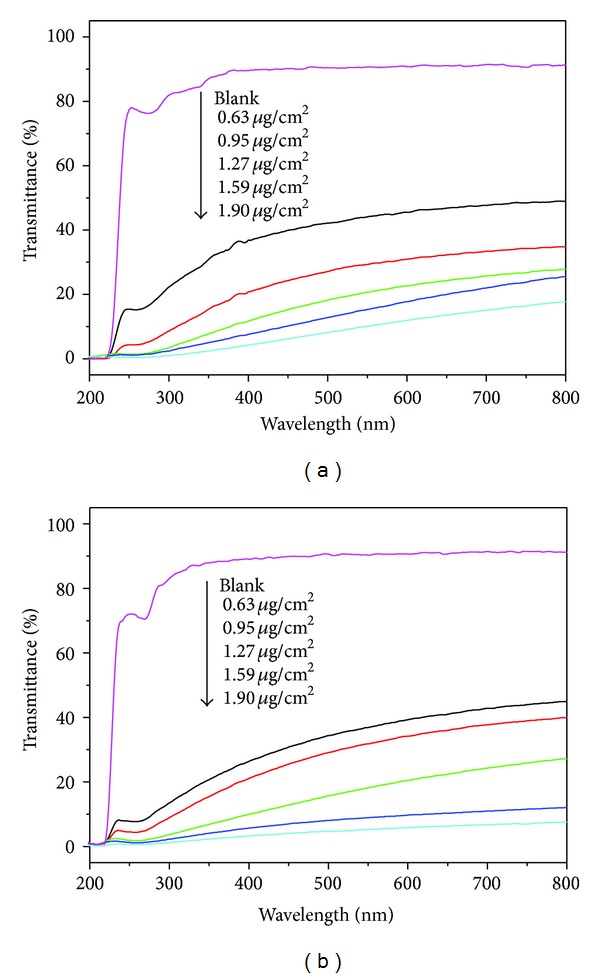
(a) UV-Vis light transmitted spectra of (a) RGO@PVA and (b) RGO@PDMS composite films.

As is well known, graphene is a good transparent material. The transmittance of the mechanically exfoliated graphene is up to 97.7%. Herein, we investigated the optical transmittance spectra of these fabricated RGO@polymer films and present them in Figures 5(a) and 5(b). We find that both the transparencies of PVA and PDMS are dramatically decreased after loading RGO on film, even though the thickness of RGO is only about 0.24 *μ*m. This is because the optical transmittance of graphene-based materials is highly dependent on the their crystal quality and the number of graphene layers [26]. Generally, the graphene sheets prepared by the reduction of GO contain many defects in chemical and crystal structure. Combining with the layers' overlapping in the film assemblage, the optical property of RGO@polymer films is inferior to pristine graphene. In spite of this, the transmittance of composite films in the visible region is still up to 45% (thickness 0.24 *μ*m), which can satisfy the requirements of certain semitransparent applications. Notably, the films possess very strong absorbance in the ultraviolet region, which would make them potential candidates for anti-UV application. 

**Figure 6:**
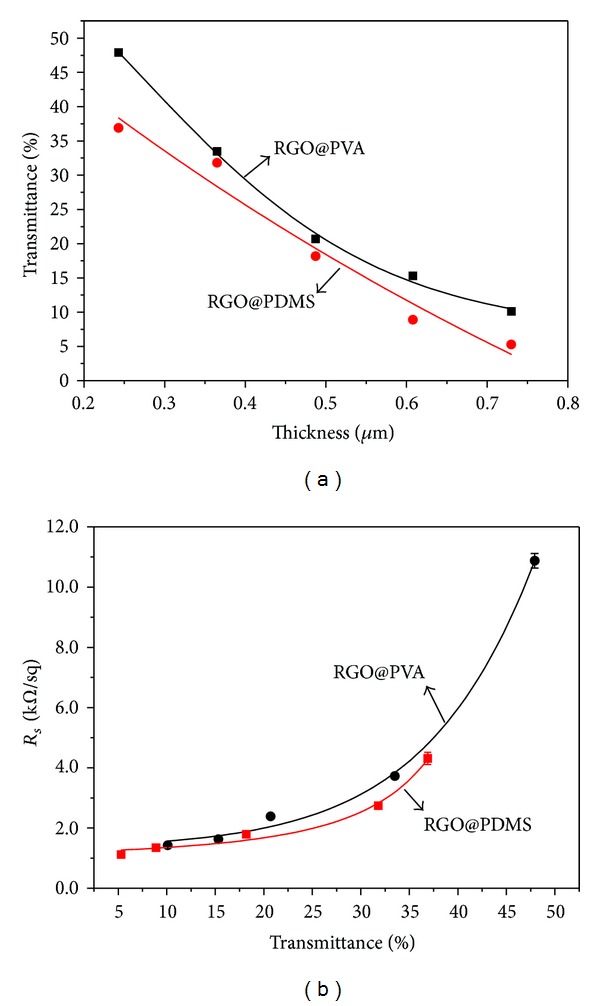
(a) Optical transmittance at 550 nm of the composite films with different thicknesses of RGO; (b) sheet resistance versus optical transmittance at 550 nm of RGO@PVA and RGO@PDMS composite films.

Figure 6(a) shows that the optical transmittance at 550 nm of composite films is decreased as the load of RGO increased. Additionally, the sheet resistance increased as optical transmittance at 550 nm decreased (Figure 6(b)). Thus, at our convenience, we could control the transmittance and sheet resistance of composite films obtained by adjusting the load of RGO. All of these results demonstrate that RGO dispersion in ethanol/water prepared via *γ*-ray irradiation is very feasible for the preparation of RGO@polymer composite films. Predictably, composite films with specific sheet resistance and transparency would make them potential materials for solar cells [27], electrical circuits [28], and antistatic coating [8, 15].

## 4. Conclusions

In conclusion, the reduced GO dispersion prepared by *γ*-ray irradiation was successfully used to prepare conductive RGO@polymer composite films. The conductivity and transmittance of films are relative to the thickness of RGO. When the thickness of RGO is about 0.24 *μ*m on PVA, the transmittance at 550 nm of RGO@PVA film reaches up to 45% plus with 10 kΩ/sq of sheet resistance. The films show good conductivity and a specific transmittance, which offers potential for use in electrical applications, optical devices, and antistatic application. Beyond that, the UV-Vis absorption spectra show that the films possess a strong ultraviolet absorption property, which would make them useful as UV-shield materials.
